# Warrick score in rheumatoid-arthritis interstitial lung disease: a promising tool for assessing the extent and progression of lung involvement

**DOI:** 10.1186/s42358-025-00435-w

**Published:** 2025-01-25

**Authors:** Duygu Temiz Karadag, Sevtap Dogan, Neslihan Gokcen, Oznur Sadioglu Cagdas, Ayten Yazici, Ayse Cefle

**Affiliations:** 1 Division of Rheumatology, Department of Internal Medicine, Kocaeli University Faculty of Medicine, İzmit, Kocaeli 41380 Turkey; 2 Department of Radiology, Kocaeli University Faculty of Medicine, Kocaeli, Turkey

**Keywords:** Rheumatoid arthritis, Interstitial lung disease, Warrick score

## Abstract

**Background:**

The clinical manifestations and course of rheumatoid arthritis-associated interstitial lung disease (RA-ILD) exhibits considerable heterogeneity. In this study, we aimed to explore radiographic progression over a defined period, employing the Warrick score as a semi-quantitative measure in early RA-ILD, and to assess the associated risk factors for progression.

**Methods:**

RA-ILD patients underwent consecutive Warrick scoring based on initial high-resolution computed tomography (HRCT) at diagnosis and the first follow-up. Associations between Warrick scores, pulmonary function tests, and patient characteristics were analyzed. The ROC curve assessed the predictive performance of the Warrick score change rate for ILD progression, while multivariable logistic regression analysis identified risk factors for progression.

**Results:**

Significant correlations were found between Warrick scores and age at RA-ILD diagnosis, age at ILD diagnosis, and baseline DAS28-ESR. For the severity score, correlations were *r* = 0.359, *r* = 0.372, and *r* = 0.298 (*p* = 0.001, *p* < 0.001, *p* = 0.014, respectively); for the extent score, *r* = 0.364, *r* = 0.318, and *r* = 0.255 (*p* = 0.001, *p* = 0.005, *p* = 0.038, respectively); and for the total score, *r* = 0.376, *r* = 0.367, and *r* = 0.280 (*p* < 0.001, *p* = 0.001, *p* = 0.022, respectively). Annual changes in severity, extent, and total Warrick scores showed sensitivities of 91–97% and specificities of 98% for predicting progression over a 5-year follow-up. Cut-off values were 0.0278 for the severity score (AUC 0.954), 0.0227 for extent score (AUC 0.976), and 0.0694 for total score (AUC 0.946). Warrick severity, extent, and total scores increased significantly during follow-up. Age > 50 years (OR 7.7; *p* = 0.028) and baseline usual interstitial pneumonia (UIP) pattern (OR 3.1, *p* = 0.041) were identified as risk factors for progression.

**Conclusions:**

Advanced age and UIP pattern were significant risk factors for progression. Warrick scoring may may help predict progression in RA-ILD, particularly through changes in severity, extent, and total scores. Due to the retrospective design and small sample size, further prospective studies with larger cohorts are needed to confirm these findings and validate Warrick scoring as a reliable marker for RA-ILD progression.

## Background

Rheumatoid arthritis (RA) is a systemic autoimmune disorder known for its diverse manifestations, including interstitial lung disease (ILD), which significantly impacts patient outcomes. ILD has been reported in 30–60% of RA patients, and the frequency can vary depending on the population studied, diagnostic criteria, and methods used for detection [1, 2]. ILD is one of the leading causes of death in RA patients, accounting for approximately 20% of mortality in subjects with RA [3]. The risk of death over the follow-up period was reported to be almost 3-fold higher in patients with RA-ILD than in patients with RA alone underscoring the importance of detecting the patients early, predicting the patients at risk of progression and establishing specialized treatment strategies to improve the prognosis [4–8].

The clinical manifestations and course of RA-ILD exhibits considerable heterogeneity. While the majority of patients with RA-ILD remain asymptomatic, clinically significant ILD is observed in only approximately 10% of cases [9]. One of the crucial aspects of RA-ILD is its variable progression. Some individuals may experience a slow progression of the disease, while others may undergo faster or progressive worsening [10–14]. Given the variability in the clinical course of RA-ILD, accurately predicting the progression of the lung disease is crucial for optimizing management and improving outcomes. To date, several factors, including older age, male sex, higher Disease Activity Score in 28 joints (DAS28) score, the presence of a usual interstitial pneumonia (UIP) pattern or a greater extent of fibrosis on high-resolution computed tomography (HRCT), elevated levels of rheumatoid factor (RF), or anti-cyclic citrullinated peptide (anti-CCP), lower forced vital capacity (FVC), and diffusing capacity of the lung for carbon monoxide (DLCO), have been demonstrated as predictors of progression and severity of RA-ILD in various studies [12, 15–17]. However, studies on to identify patients at risk of progression early and to establish evidence-based recommendations for screening and monitoring have been increasing in recent years.

High-resolution computed tomography (HRCT) imaging is pivotal in both identifying and characterizing ILD in RA patients [18, 19]. Beyond its capacity to detect the disease in its early stages, often before symptoms manifest, HRCT provides important information regarding disease progression. It aids in quantifying the extent of lung involvement and discerning patterns indicative of disease severity and advancement [20]. Unlike respiratory symptom exacerbations or declining pulmonary function test results, which serve as established parameters for progression, HRCT offers a more objective means of assessing disease advancement, particularly when interpreted by experienced radiologists [21]. Although guidelines recommend qualitative evaluation of HRCT images to monitor radiographic progression, significant variability in interpretation raises concerns regarding detection of deterioration. The lack of a widely accepted and adequate measurement technique to evaluate HRCT findings has limited its utility in clinical practice. To address this issue, several semi-quantitative methods have been developed, offering promising tools for standardization. Among these, the Warrick score stands out as a semi-quantitative method that evaluates the extent and severity of pulmonary damage, providing a total HRCT score. It appears to be a suitable tool for measuring changes in RA-ILD-related lung abnormalities over time [22]. By systematically grading features such as ground-glass opacities, reticulation, and honeycombing, the Warrick score offers a standardized approach to assessing the severity of lung involvement [23, 24].

Despite advancements in comprehending the trajectory of RA-ILD, uncertainties persist regarding whether lung involvement follows a time-dependent pattern or exhibits variations between early and late disease phases. This study aims to explore radiographic progression over a defined period, employing the Warrick score as a semi-quantitative measure in early RA-ILD, and to assess the associated risk factors for progression.

## Materials and methods

### Study population

We retrospectively reviewed the RA-ILD cohort, encompassing patients with follow-up chest HRCT scans between January 2009 and December 2023. The patients included in this study fulfilled the 2010 American College of Rheumatology/European League Against Rheumatism (ACR/ EULAR) classification criteria [25].

All patient information, including demographic details, HRCT images, pulmonary function tests (PFTs), and laboratory data, was extracted from medical records. Disease duration was calculated as the time between the onset of the first RA symptom and the enrollment date. Baseline disease activity was assessed using the Disease Activity Score in 28 joints-Erythrocyte Sedimentation Rate (DAS28-ESR), with scores calculated within 3 months of the HRCT scan and retrieved from patients’ medical records. Patients’ symptoms, such as cough, dyspnea, and fatigue, recorded during clinic visits coinciding with the first HRCT and follow-up HRCT times, were obtained from their medical records. Similarly, PFT results corresponding to the baseline and follow-up HRCT scans were retrieved from the patients’ records.

We included the initial scan obtained at the time of ILD diagnosis and the second scan from the first year of follow-up. In cases where the first-year follow-up scan was unavailable, we included the first interpretable follow-up scan that was technically suitable for evaluation. All patients underwent chest HRCT using a 64-detector scanner (Aquilion 64; Toshiba Medical Systems, Tokyo, Japan) while in a supine position, with full inspiration and without contrast enhancement. The scanning parameters were set at 120 kV, with variable mAs adjusted according to patient size using an automatic exposure control system, and a slice thickness of 1 mm. The images were reconstructed in a 512 × 512 matrix with 1 mm non-overlapping slices, employing a standard HRCT reconstruction algorithm. Prone scans targeting the lung bases were performed when ground-glass opacities were observed in the posterobasal subpleural region, aimed at excluding gravitational opacities. All images were viewed with a window level of − 600 Hounsfield units and a width of 1,600 Hounsfield units.

We excluded the patients with non-fibrotic abnormalities, including pneumonia, pulmonary edema, pulmonary thromboembolism, severe left ventricular failure, and severe emphysema by visual assessment based on the HRCT images. In case of doubt about the acute abnormal findings (infection, etc.), pulmonary involvement was confirmed with supportive findings in follow-up HRCTs. Patients with a diagnosis of other respiratory disorders, such as asthma or chronic obstructive pulmonary disease, malignancy, and significant pulmonary hypertension defined by previous clinical or echocardiographic evidence of significant right heart failure or requiring parenteral therapy were also excluded. The exclusion was made to prevent bias due to the non-ILD-related fibrosis in the evaluation of HRCT images.

### Image interpretation

All CT images underwent re-evaluation and interpretation by a thoracic radiologist (S.D.) with 15 years of experience in thoracic radiology. The radiologist, blinded to the clinical data, independently diagnosed ILD based on radiological findings such as reticulation, traction bronchiectasis, honeycomb cysts, ground-glass opacities or airspace consolidation, and other interstitial lung abnormalities. The pattern of ILD was classified according to official guidelines, with pulmonary fibrosis patterns on HRCT further categorized into definite UIP with honeycombing, probable UIP, non-specific interstitial pneumonia (NSIP), and organizing pneumonia (OP) patterns, following standard radiological terminology [21, 26]. According to ATS/ERS/JRS/ALAT Clinical Practice Guideline progressive lung fibrosis was characterized by the presence of at least two of the following three criteria within the past year, without any alternative explanation: (I) worsening respiratory symptoms, (II) physiological evidence of disease progression (manifested as either an absolute decline in FVC > 5% predicted within 1 year of follow-up or an absolute decline in DLCO > 10% predicted within 1 year of follow-up), and (III) radiological evidence of disease progression [21]. According to this definition, patients with worsening respiratory function test results or respiratory symptoms, in addition to radiological progression, were considered to have progressive disease. Due to the retrospective design of our study, it was not possible to obtain pulmonary function test data for all patients. Therefore, the presence of newly developed or worsening crackles on physical examination, as well as newly developed or worsening dyspnea or cough that could not be attributed to another cause, were used as clinical criteria to determine worsening respiratory symptoms. In a small number of patients without data to meet the pulmonary function test or clinical criteria, the radiologist’s assessment of significant radiological progression was taken into consideration.

### HRCT scoring

**Fig. 1:**
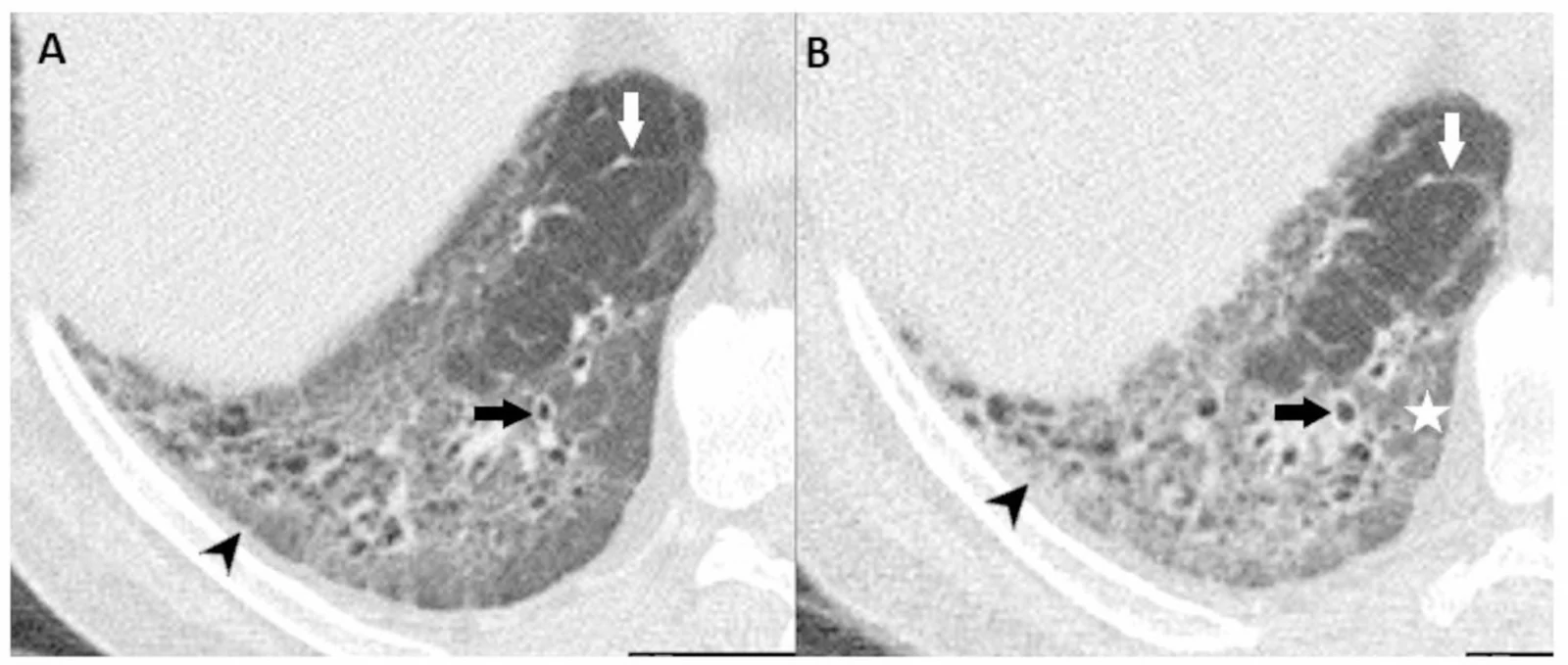
**a** and **b**: In a 70-year-old male patient with known rheumatoid arthritis, a fibrosing NSIP pattern is observed in high-resolution computed tomography scans taken at 7-year intervals. In axial sections taken from the lung bases, irregularities are observed to become evident in the subpleural area consistent with fibrotic changes (black arrowheads). Interlobular septal lines are seen as tiny lines outlining secondary pulmonary lobules forming polygonal shapes in both scans (white arrows). They are more prominent in the later section in figure b due to progressive fibrosis. In consecutive sections, an increase in ground- glass opacities (asterix) are visible, as well as traction bronchiectasis (black arrows)

The initial and follow-up HRCT images were assessed using the semiquantitative method proposed by Warrick et al. This scoring system identifies five elementary lesions, including ground-glass opacities, irregularities in the pleural margins, septal lines, honeycombing, and subpleural cysts, each rated from 1 to 5 (Fig. 1a/b and 2a/b). These ratings contribute to a severity score, which is the sum of the individual scores for each observed lesion on HRCT images. Consequently, the severity score ranges from 0 (no elementary lesions) to 15 (all elementary lesions present). Subsequently, the extent score for each patient was determined based on the number of segments involved for each elementary lesion. Lesions were categorized into scores of 1 to 3, where 1 indicates involvement in 1–3 segments, 2 indicates involvement in 4–9 segments, and 3 indicates involvement in more than 9 segments. The Warrick scoring criteria are presented in Table 1 [27]. The total Warrick score was calculated by combining the severity and extent scores, resulting in a range between 0 and 30 [22].

**Fig. 2:**
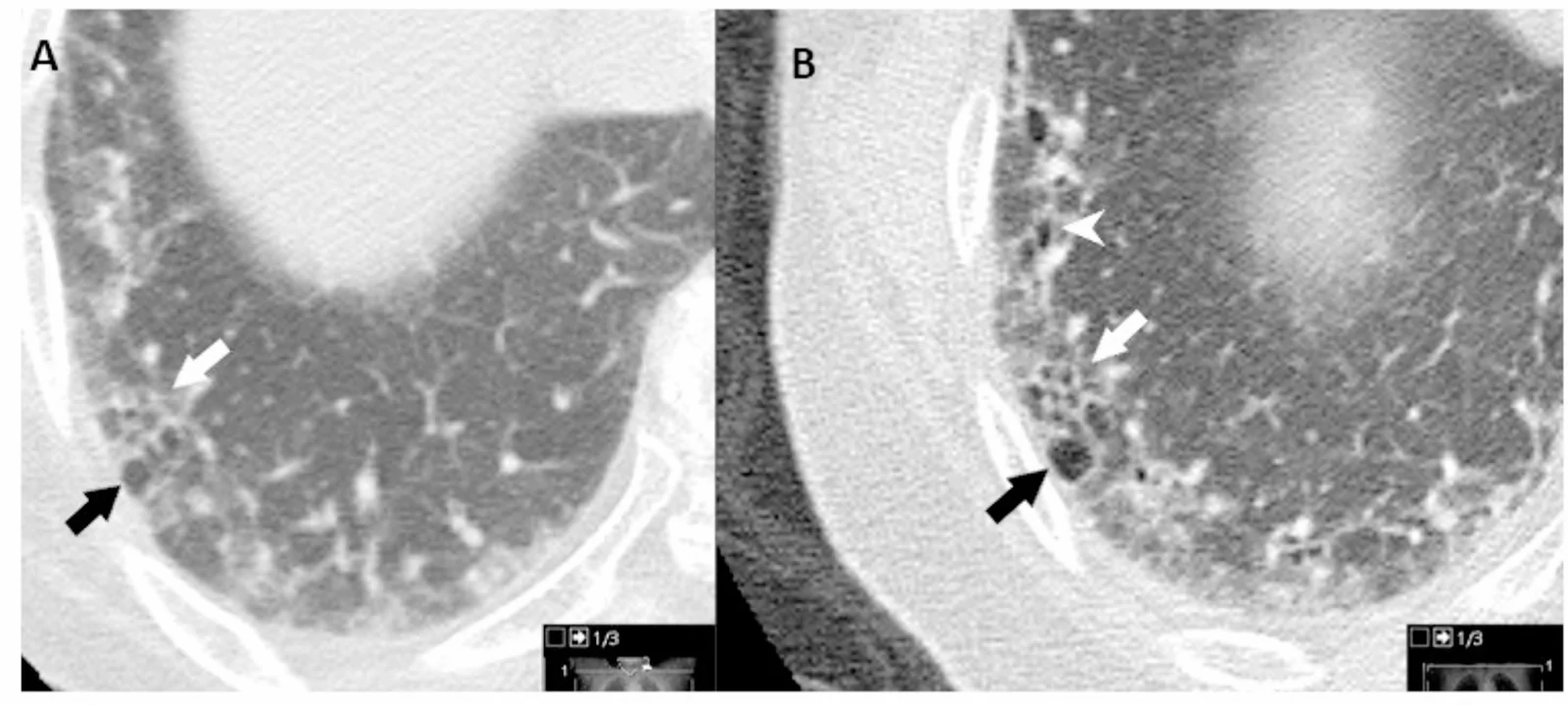
**a** and **b**: In a 86-year-old female patient with known rheumatoid arthritis, a UIP pattern is observed in computed tomography scans at 2-year intervals. High-resolution computed tomography image at the right pulmonary bases shows honeycombing seen as small, thick-walled cystic spaces arranged in multiple layers in the subpleural region which becomes more prominent in the later scans in figure **b** (white arrows). There is also a large subpleural cyst, which increases in size over time (black arrows). In figure **b**, traction bronchiectasis also shows progression (arrowhead)

**Table 1:** Criteria used for calculating the Warrick score

Lesions and lung segments	Score
Parenchymal abnormalities	Disease severity score
Ground-glass opacities	1
Irregularities in the pleural 2 margins	2
Septal/subpleural lines	3
Honeycomb lung	4
Subpleural cysts	5
Number of lung segments	Disease extent score
1–3	1
4–9	2
> 9	3

### Additional semi-quantitative parameters

We hypothesized that given the variable nature of disease progression across different time intervals, evaluating progression per unit time could offer a valuable method for assessing patients with longer durations of ILD. However, the absence of a standardized definition for progression rate in the literature prompted us to devise a calculation method. We computed a change rate to measure progression per unit time, termed the “Warrick score change rate,” between two consecutive HRCT scans. This involved subtracting the initial score from the last Warrick score calculated on two consecutive HRCT scans and dividing the difference by the time (in years) between the two CT scans.

### Statistical analysis

Descriptive statistics for clinical and demographic characteristics of the patients were presented as frequency and percentage (%) for categorical variables and mean with standard deviation (mean ± SD) or median with interquartile range (median [IQR = Q3–Q1]) depending on the distribution of the continuous variables. The distribution normality of numerical data was evaluated visually and with the Shapiro-Wilk test.

Spearman or Pearson correlation analysis was performed according to the distribution of numerical data to evaluate the relationship between the Warrick scores derived from the baseline HRCT images and the age at RA diagnosis, age at ILD diagnosis, disease duration at ILD diagnosis, DAS28-ESR at RA diagnosis, FVC and DLCO at ILD diagnosis, RF titer and anti-CCP titers. The Wilcoxon Signed Ranks Test was used to compare the change in the Warrick scores and HRCT after time intervals. We used Mann Whitney U or independent samples t-test for continuous variables and a Chi-squared test for categorical variables to analyze the distribution of demographic, disease-related clinical, laboratory, and radiological data in patients with and without progression.

The receiver operating characteristic (ROC) curve was used to evaluate the performance of Warrick score change rates and determine the presence of patients with ILD progression detected by visual evaluation. We conducted multivariable logistic regression analysis to identify the risk factors associated with the progression of ILD.

Statistical analyses were performed using the SPSS version 20.0 software package (IBM Inc., Chicago, IL, USA). Two-sided p values less than 0.05 were considered statistically significant (*p* < 0.05).

## Results

We enrolled 77 patients, comprising 45 women and 32 men, with a mean age of 60.61 ± 8.68 years. The mean duration of RA was 8.41 ± 7.07 years, while the mean duration of ILD was 5.36 ± 3.5 years. All demographic and clinical characteristics were recorded at the time of ILD diagnosis (Table 2). Patients with progression were classified based on radiology, pulmonary function tests, and clinical criteria in 6 patients; radiology and clinical criteria in 17 patients; radiology and pulmonary function tests in 8 patients; and based on the radiologist’s judgment of significant radiological progression in 3 patients.

**Table 2:** Baseline demographic, clinical, and laboratory features at the time of ILD diagnosis

		Mean ± SD or Median [Q1-Q3]
Age at RA diagnosis		54.1 ± 10.7
Age at ILD diagnosis		60.61 ± 8.68
Gender (female) (n, %)		45/77
Disease duration (years)		8.41 ± 7.07
DAS28-ESR		3.32 ± 1.24
Swollen joint (n, %)		-
Tender joint (n, %)		-
Cough (n, %)		22/67
Dyspnea (n, %)		17/67
Velcro crackles (n, %)		24/66
Smoking (n, %)		
	Never	39/74
	Active	24/74
	Quit	11/74
Smoking (pack-year)		22.55 ± 15.51
ESR		32.13 ± 25.57
CRP		0.69 ± 0.75
FVC (%)		87 [74.5–99.5]
DLCO (%)		62 [45.75–84.5]
Anti-RF (positive) (n, %)		63/76
Anti-CCP (positive) (n, %)		56/75
Interval between consecutive HRCT		5.36 ± 3.5

ILD, interstitial lung disease; DAS28-ESR, Disease Activity Score-28; ESR, erythrocyte sedimentation rate; CRP, C-Reactive Protein; FVC, forced vital capacity; DLCO, diffusing capacity of carbon monoxide; anti-RF, anti- Rheumatoid factor; anti-CCP, anti-cyclic citrullinated peptide

When analyzing the correlation between baseline Warrick scores obtained from baseline HRCT images and demographic and clinical characteristics, we observed a positive correlation between severity score and age at RA diagnosis, age at ILD diagnosis, and DAS28-ESR (*r* = 0.359, *p* = 0.001; *r* = 0.372, *p* = < 0.001; and *r* = 0.298, *p* = 0.014, respectively). Similarly, extent score correlated positively with age at RA diagnosis, age at ILD diagnosis, and DAS28-ESR (*r* = 0.364, *p* = 0.001; *r* = 0.318, *p* = 0.005; and *r* = 0.255, *p* = 0.038, respectively). Moreover, total score exhibited positive correlations with age at RA diagnosis, age at ILD diagnosis, and DAS28-ESR (*r* = 0.376, *p* = < 0.001; *r* = 0.367, *p* = 0.001; and *r* = 0.280, *p* = 0.022, respectively) (Table 3).

**Table 3:** Correlation of baseline disease-related parameters with Warrick severity, extent, and total score measured at initial HRCT

		Age^*^	Age^**^	Disease duration	DAS28-ESR	Smoking (pack/year)	FVC (%)	DLCO (%)	Anti-RF titer	Anti-CCP titer
Severity score	*r*	0.359	0.372	-0.001	0.298	0.196	0.229	-0.011	-0.068	0.024
	*p*	0.001	< 0.001	0.997	0.014	0.309	0.272	0.952	0.583	0.857
Extent score	*r*	0.364	0.318	-0.082	0.255	0.255	0.082	-0.083	-0.036	-0.010
	*p*	0.001	0.005	0.482	0.038	0.181	0.698	0.652	0.770	0.940
Total score	*r*	0.376	0.367	-0.037	0.280	0.241	0.160	-0.057	-0.071	0.022
	*p*	< 0.001	0.001	0.754	0.022	0.208	0.445	0.757	0.566	0.873

Age*, age at diagnosis of RA; Age**, age at diagnosis of ILD. (All the other parameters represent the baseline values). DAS28-ESR, Disease Activity Score-28; FVC, forced vital capacity; DLCO, diffusing capacity of carbon monoxide; anti-RF, anti- Rheumatoid factor; anti-CCP, anti-cyclic citrullinated peptide

We detected progression in 43 out of 77 patients (55.8%) through ATS/ERS/JRS/ALAT Clinical Practice Guideline. No differences were observed between progressors and non-progressors regarding baseline demographic, disease-related clinical, laboratory, and radiological data (Table 4).

**Table 4:** Distribution of baseline demographic, disease-related clinical, laboratory and radiological data in RA-ILD patients with and without progression

	Progressors *N* = 43	Non- Progressors *N* = 34	*p*
Gender (female)	26/43 (60.5%)	19/34 (55.9%)	0.816
Probable UIP patern	23/43 (53.5%)	24/34 (70.6%)	0.161
Definite UIP patern with honey combing	6/43 (14%)	9/34 (26.5%)	0.247
NSIP	7/43 (16.3%)	6/34 (17.6%)	1.000
Cough	10/35 (23.3%)	32/32 (37.5%)	0.603
Dyspnea	10/35 (23.3%)	7/32 (20.6%)	0.584
Velcro crackles	12/34 (27.9%)	12/32 (35.3%)	1.000
Smoking	11/38 (25.6%)	11/26 (32.4%)	0.542
Anti-CCP (positive)	35/42 (81.4%)	28/34 (82.4%)	0.793
Anti-RF (positive)	32/42 (74.4%)	24/33 (70.6%)	1.000
Worsening respiratory symptoms	10/33 (23.3%)	10/28 (29.4%)	0.786
Age at RA diagnosis	55.34 ± 11.61	52.90 ± 9.7	0.529
Disease duration at ILD diagnosis	6.34 ± 6.4	6.93 ± 6.87	0.687
Age at ILD diagnosis	61.3 ± 9.27	59.7 ± 7.91	0.593
Baseline Warrick Severity score	4.95 ± 2.72	5.65 ± 3.02	0.331
Baseline Warrick Extent score	4.84 ± 2.62	5.38 ± 2.92	0.548
Baseline Warrick Total score	9.79 ± 5.13	11.03 ± 5.70	0.428
Baseline DAS28-ESR	3.57 ± 1.34	3.04 ± 1.08	0.117
Smoking (pack/year)	21.80 ± 16.10	23.36 ± 14.51	0.949
FVC % at ILD diagnosis	88.81 ± 14.98	83.44 ± 18.82	0.598
DLCO % at ILD diagnosis	64.38 ± 23.94	63.88 ± 19.51	0.949
Anti-RF titer	348 ± 681	361 ± 748	0.786
Anti-CCP titer	154 ± 207	290 ± 389	0.141

UIP, usual interstitial pneumonia; NSIP, nonspecific interstitial pneumonia; anti-RF, anti- Rheumatoid factor; anti-CCP, anti-cyclic citrullinated peptide; ILD, interstitial lung disease; DAS28-ESR, Disease Activity Score-28; FVC, forced vital capacity; DLCO, diffusing capacity of carbon monoxide

We assessed whether there was a change from baseline in the follow-up CT characteristics. The Warrick-severity score, Warrick- extent score, and Warrick-total score were increased significantly at the end of a median time of 5 years (6.48 ± 3.75 vs. 5.26 ± 2.86, *p* < 0.001; 5.94 ± 3.21 vs. 5.08 ± 2.75, *p* = 0.001; 12.42 ± 6.68 vs. 10.34 ± 5.39, *p* < 0.001, respectively**)**. We also found that a definite UIP pattern with honeycombing was higher at the end of follow-up HRCT (32.5% vs. 19.5%, *p* = 0.002). There were no differences in the frequency of probable UIP, NSIP, or OP patterns (Table 5).

**Table 5:** Change in the Warrick score and fibrosis paterns between consecutive HRCT images

	1.HRCT (*n* = 77)	2.HRCT (*n* = 77)	*p*
Warrick-severity score	5.26 ± 2.86	6.48 ± 3.7	< 0.001
Warrick- extent score	5.08 ± 2.75	5.94 ± 3.21	0.001
Warrick-total score	10.34 ± 5.39	12.42 ± 6.68	< 0.001
Definite UIP patern with honey combing	15 (19.5%)	25 (32.5%)	0.002
Probable UIP patern	32 (41.6%)	26 (33.8%)	0.058
NSIP	13 (16.9%)	12 (15.6%)	1
Nodule	7 (9.1%)	10 (13%)	0.25
Organizing pneumonia	3 (3.9%)	1 (1.3%)	0.625

HRCT, High-resolution computed tomography; UIP, usual interstitial pneumonia; NSIP, nonspecific interstitial pneumonia

We conducted a binary logistic regression analysis to determine the predictive factors for progression. The model included variables such as being over 50 years old, duration of the disease at the time of ILD diagnosis, and the presence of a UIP pattern in the initial HRCT as predictor variables. According to the model we constructed, we observed a higher risk of disease progression in individuals over 50 years old compared to younger patients (OR 7.7, 95% CI [1.25–46.91]; *p* = 0.028) and in patients with a UIP pattern at baseline CT compared to those without (OR 3.1, 95% CI [1.10–9.57]; *p* = 0.041). The Warrick extent, severity, or total scores in the baseline HRCT did not contribute to predicting progression.

**Fig. 3:**
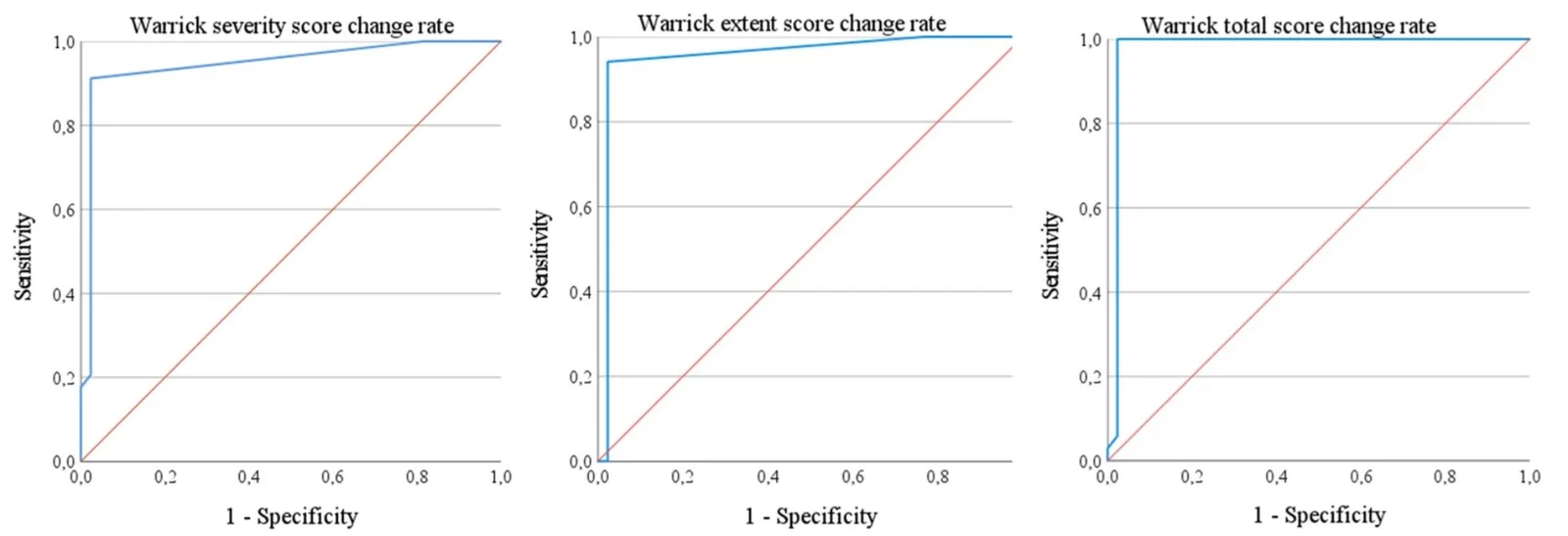
The receiver operating characteristic (ROC) curve to evaluate the performance of Warrick score change rates to determine the progression of patients with ILD

After analyzing the Warrick score change rates, we found that the Warrick severity score increased by 0.21 ± 0.62; the Warrick- extent score was 0.12 ± 0.65; the Warrick-total score was 0.32 ± 1.17 annually. ROC curve analysis was conducted to assess the utility of the rate of change of the Warrick score as a parameter for identifying patients with progression over an average period of 5 years. The results of ROC curve analysis for evaluating the performance of Warrick score change rates revealed excellent results for Warrick-severity score change rate, Warrick-extent score change rate, and Warrick-total score change rate (AUC 0.946, 0.954, 0.976 respectively, and *p* < 0.001 for all) (Fig. 3). A Warrick-severity score change rate of 0.0277 showed a sensitivity of 91% and specificity of 98%; an extent score change rate of 0.0227 showed a sensitivity of 94% and specificity of 98%, and a total score change rate of 0.0694 showed a sensitivity of 97% and specificity of 98%.

## Discussion

In this study, we aimed to elucidate the role of Warrick scoring in evaluating the progression of RA-ILD patients. Our findings revealed significant associations between Warrick scores and clinical parameters such as age at RA and ILD diagnosis and baseline DAS28-ESR. The most significant finding of our study was the promising predictive capability of Warrick score change rates in identifying patients with progression within the initial 5 years of ILD.

The results of our study revealed a correlation between the Warrick score and the age at RA diagnosis, age at ILD diagnosis, and baseline DAS28-ESR. Consistent with our findings, a correlation between age and the Warrick score was revealed in a cross-sectional study. Additionally, it demonstrated positive correlations with dyspnea, cough, and RF titer and a negative correlation with DLCO% [28]. Previous studies have recognized advanced age and high disease activity as risk factors for ILD development in RA patients. Furthermore, it has been reported that the UIP pattern, linked to poor prognosis, was more frequently observed in older patients with a longer duration of the disease [29]. These correlations highlight the potential utility and concordance of Warrick scoring with existing data on disease course and severity.

RA-associated interstitial lung disease (RA-ILD) can vary in clinical course, from rapid progression to stability or even improvement [30]. Progression patterns may differ at various stages, raising questions about directly applying criteria developed for idiopathic pulmonary fibrosis (IPF), which tends to progress more aggressively than connective tissue disease-related ILDs [21]. The definition of progression in connective tissue diseases remains under research. Recently, several definitions for progressive fibrosing ILD have emerged, primarily relying on pulmonary function tests (PFTs), except for the ATS/ERS/JRS/ALAT guidelines, which also consider clinical and radiographic findings. For example, the RELIEF trial defined progression as an annual decline in FVC > 5%, while the TRAIL1 trial defined it as a ≥ 10% relative decline in FVC or a 5–10% decline combined with a ≥ 15% decline in DLCO within one year [31, 32]. The updated ATS/ERS/JRS/ALAT guidelines now define progressive fibrosing phenotypes (PPFs) based on radiological, physiological, and clinical evidence of progression within one year [21]. However, these definitions may overlook long-term fluctuations in disease trajectory. In contrast, recent SSc-ILD treatment guidelines have removed time criteria from their definition of progression, highlighting that this area remains under ongoing research and is subject to change as new insights emerge [33].

Taking into account previous definitions and the longitudinal nature of disease fluctuation, we investigated the rate of change in the Warrick score over an average of five years in early ILD in our study. Based on our analysis, we found that an annual change of 0.0277 in the Warrick severity score, 0.0227 in the extent score, and 0.0694 in the total score effectively identified patients with progression over an average of 5 years. Our results demonstrated that the cut-off values we defined for annual changes in severity, extent, and total Warrick scores exhibited high sensitivity and specificity for predicting progression. However, when interpreting our results, it’s important to consider that the time interval between consecutive HRCTs can influence the rate of score changes, especially in a disease like RA that exhibits a variable course. Examining rates of change over shorter intervals, such as yearly assessments, would enhance our understanding of this matter. However, due to our utilization of the first interpretable follow-up HRCT images in our study, we were unable to conduct this analysis.

One notable finding of our study was the relatively low prevalence of symptoms among RA-ILD patients, with only 28.60% experiencing cough, 22.10% reporting dyspnea, and 31.20% exhibiting velcro crackles. In the study by D. Chai et al., nearly half of the RA-ILD patients, encompassing preclinical and clinical ILD, did not exhibit respiratory symptoms or signs. Notably, in patients with preclinical ILD, this rate reached 83.3% [34]. While routine screening for ILD is not generally recommended in systemic autoimmune rheumatic diseases, it is conditionally advised for individuals at an increased risk of developing ILD [35]. The absence of a strong recommendation for routine ILD screening may result in delayed diagnosis for patients experiencing progression, as well as potential overdiagnosis of individuals with a natural, non-progressive, subclinical course. Our study may help mitigate uncertainties within this gray area.

We identified age over 50 and a UIP pattern on baseline CT as risk factors for progression. Male gender, higher DAS28-ESR levels, HRCT-documented UIP-like fibrotic pattern, and higher baseline HRCT scores, were identified as poor prognostic factors in RA-ILD [36, 37]. Additionally, older age at ILD diagnosis and UIP pattern were identified as risk factors for acute exacerbations [38]. While the risk factors reported in different studies are compatible, the primary reason for the variations among them may be attributed to the different definitions of progression used in the studies. For instance, in some studies, the definition of progression is based solely on worsening in pulmonary function tests, while in others, it includes deterioration in both pulmonary function tests and chest CT findings [36, 37]. Another reason for the variations in the reported progression risk factors could be attributed to differences in the patient populations across studies. In our study, the average follow-up duration of RA-ILD patients was 5 years, contrasting with the 19-month average reported by Chai et al. [34]. The characteristics of the study populations should be taken into account when interpreting the risk factors for progression.

Our study revealed no differences between progressors and non-progressors concerning baseline demographic, clinical, laboratory, and radiological features. In this regard, the literature provides data that both aligns and diverges from our findings. For instance, Chai et al. reported that progressors tended to be older (> 60 years), smokers, individuals with diabetes mellitus, and those experiencing dyspnea and velcro crackles. Additionally, progressors showed elevated levels of various biomarkers, including DAS28- erythrocyte sedimentation rate (DAS28-ESR), C-reactive protein (CRP), and positive anti-CCP antibody. In contrast, Lee J et al. found that most clinical characteristics were not significantly different between patients with and without progressive pulmonary fibrosis, regardless of the diagnostic criteria used. These discrepancies may stem from variations in patient cohorts and underscore the unique nature of RA-ILD. When interpreting the results, it is essential to consider factors such as the predominant clinical characteristics of RA patients in the cohorts, as well as variables like age, disease duration, and the subclinical or clinical nature of RA-ILD. Chai et al. also exhibited higher baseline and follow-up HRCT scores compared to non-progressors. In our study, we did not find a relationship between baseline severity and extent scores and disease progression. While fibrosis scores, typically calculated as the sum of honeycombing and reticulation scores, are often associated with progression in RA-ILD [39], our study included lesions related to both fibrosis and alveolitis (e.g., ground-glass opacity). This may explain the lack of association with progression, as the HRCTs we analyzed were from patients with early-stage RA-ILD, where alveolitis is more prominent than fibrosis. Alveolitis, characterized by inflammation, may represent a more reversible or fluctuating process, whereas fibrosis, particularly in the UIP pattern, reflects permanent and progressive damage. The association of the UIP pattern, which indicates heterogeneous fibrosis in the lung parenchyma, with progression in our study further supports this distinction.

Our study has several limitations. Firstly, it was a single-center, retrospective cohort study with a small sample size. Secondly, although RA-ILD patients underwent regular follow-up, some of the first follow-up HRCTs which were performed a long period of time were not eligible for evaluation because of the technical issues. This forced us to evaluate the images of HRCT conducted several years after the initial CT scan. One of the limitations of our study is the inability to obtain detailed information on patient symptoms and physical examination findings due to its retrospective design. Lastly, we did not assess the impact of medications, which likely have a significant role in ILD progression.

## Conclusions

This study highlights the potential role of Warrick scoring in predicting progression in RA-ILD patients, particularly through changes in severity, extent, and total scores. Additionally, baseline advanced age and the presence of a UIP pattern were identified as significant risk factors for progression, aligning with previous studies on RA-ILD. Our findings suggest that the rate of change in Warrick scores over time may help identify patients at risk of progression within the first five years of ILD. However, the retrospective nature of our study and the small sample size limit the generalizability of these results. Further prospective studies involving larger cohorts and incorporating regular HRCT and PFT assessments are needed to validate these preliminary observations and establish Warrick scoring as a robust marker for disease progression in RA-ILD.

## Data Availability

The datasets used and/or analysed during the current study are available from the corresponding author on reasonable request.

## Abbreviations

RA: Rheumatoid arthritis
ILD: Interstitial lung disease
HRCT: High-resolution computed tomography
UIP: Usual interstitial pneumonia
FVC: Forced vital capacity
DLCO: Diffusing capacity of the lung for carbon monoxide
DAS28-ESR: Disease Activity Score in 28 joints-Erythrocyte Sedimentation Rate
PFTs: Pulmonary function tests
NSIP: Non-specific interstitial pneumonia
ROC: Receiver operating characteristic
OP: Organizing pneumonia
RF: Rheumatoid factor
Anti-CCP: Anti-cyclic citrullinated peptide
ACR/ EULAR: American College of Rheumatology/European League Against Rheumatism
ESR: Erythrocyte sedimentation rate
CRP: C-reactive protein
